# 505 Fusarium Isolates in Burn-injured Patients: Clinical Characteristics and Susceptibility Patterns

**DOI:** 10.1093/jbcr/irae036.140

**Published:** 2024-04-17

**Authors:** Sierra R Young, Robyn N Stoianovici, Erin L Louie

**Affiliations:** UC Davis Health, Sacramento, CA; UC Davis Health, Sacramento, CA; UC Davis Health, Sacramento, CA

## Abstract

**Introduction:**

Fusarium species are ubiquitous in the environment and can cause opportunistic infections in burn-injured patients. Patients with larger burns, prolonged lengths of stay, concomitant antimicrobial use and invasive lines are at higher risk for Fusarium infections. Little data exists on clinical characteristics and in vivo antifungal susceptibilities in burn-injured patients.

**Methods:**

All positive Fusarium species cultures were retrospectively identified between November 2017 to June 2023 at a regional burn center. Demographic, clinical and microbiologic susceptibility data was collected from the electronic medication record. Descriptive statistics were used to describe the cohort.

**Results:**

A total of 18 patients had positive Fusarium wound cultures during the study period. The average age was 38.4 ± 11.9 years. The average %TBSA was 54.5 ± 23.4 % and R-Baux score of 92.9 ± 29.3. The most common mechanism of injury was thermal burn (17 patients, 94%). This cohort experienced prolonged ICU (68.9 ± 31.9 days) and hospital (73.6 ± 38.2 days) lengths of stay. The average time from burn injury to positive Fusarium cultures was 19.6 ± 9.0 days. Mechanical ventilation was prolonged (46.1 ± 23.3 days). The average central line duration prior to Fusarium infection was 9.8 ± 10.2 days. Susceptibility data was obtained for voriconazole in 15 patients and for amphotericin in 14 patients. For voriconazole, 5/15 tested isolates had an MIC of 4, 1/15 had an MIC of 8, and 9/15 (60%) isolates returned with MIC ≥ 16. A majority (13/14, 93%) of the MICs tested for amphotericin were ≤ 1. Patients were taken to the OR for source control in all cases. Ten of 18 patients with Fusarium positive infections survived to hospital discharge (55%). The most common cause of death was multi-system organ failure and sepsis (88%). Recorded adverse drug events possibly due to voriconazole and posaconazole included transaminitis (4 cases), QTc prolongation (1 case), and hallucinations (1 case). Amphotericin was suspected of contributing to an acute kidney injury in 1 case.

**Conclusions:**

This case series reports the clinical characteristics and susceptibility data on Fusarium isolates in a burn-injured population. This cohort was critically ill based on the %TBSA, R-Baux score, and high rate of mechanical ventilation. The MIC results obtained for voriconazole demonstrated a majority of isolates with relatively high MIC values ≥ 16. A majority of the MICs for amphotericin were < 1. It is important to note that no established breakpoints exist for Fusarium species and clinical outcomes are dependent on a variety of factors beyond the susceptibility data.

**Applicability of Research to Practice:**

Empiric double coverage with voriconazole and amphotericin or amphotericin monotherapy should be considered when Fusarium is a suspected primary pathogen.

